# Unveiling low level viremia in chronic hepatitis B: challenges and new horizons

**DOI:** 10.3389/fcimb.2025.1663858

**Published:** 2025-11-19

**Authors:** Bingqing Wang, Tian Zeng, Wen Yin, Chengrui Ren, Yuting Chen, Liqin Qian, Peng Huang, Chuanlong Zhu, Ming Yue

**Affiliations:** 1 Department of Infectious Diseases, The First Affiliated Hospital with Nanjing Medical University, Nanjing, Jiangsu, China; 2 Department of Epidemiology, Center for Global Health, School of Public Health, Nanjing Medical University, Nanjing, Jiangsu, China

**Keywords:** chronic hepatitis B, low level viremia, nucleos(t) ide analogs, peginterferon, fatty liver disease, clinical remission

## Abstract

Low-level viremia (LLV) in chronic hepatitis B (CHB) represents a significant challenge and area of interest in current clinical management. While nucleos (t)ide analogs (NAs) have demonstrated substantial efficacy in suppressing hepatitis B virus (HBV) replication, the application of highly sensitive detection methods has revealed that some treated patients continue to exhibit persistent or intermittent low-level viremia (HBV DNA: 12–2000 IU/mL). The mechanisms underlying LLV involve a synergistic interplay between host immune response deficiencies and HBV covalently closed circular DNA (cccDNA) persistence. Furthermore, the complex regulation of LLV is influenced by metabolic-associated steatotic liver disease (MASLD). Limitations in the clearance of cccDNA by current antiviral regimens also contribute to this phenomenon. LLV may elevate the risk of liver fibrosis progression, hepatocellular carcinoma (HCC), and end-stage liver disease. Current management strategies emphasize optimizing antiviral regimens, such as switching to tenofovir alafenamide (TAF) or combining therapies with pegylated interferon-alpha (Peg-IFN-α). Enhanced dynamic monitoring, including high-sensitivity HBV DNA assays and quantitative hepatitis B surface antigen (HBsAg) measurements, is also crucial. Moreover, exploring combination therapies involving immunomodulation and hepatocyte regeneration is warranted. Further research should integrate multi-omics technologies with prospective cohort studies to elucidate the host-virus interaction network in LLV. This will allow for the validation of synergistic effects between metabolic interventionsand immunotherapy, thereby advancing personalized precision medicine. This review systematically synthesizes the epidemiological characteristics, pathogenesis, influencing factors, prognosis, and clinical management advancements of LLV, aiming to provide novel perspectives for optimizing therapeutic strategies and translational research.

## 1 Introduction

Hepatitis B virus (HBV) infection is a global health concern. According to the World Health Organization (WHO), an estimated 296 million individuals worldwide are chronic carriers of HBsAg. In 2022, approximately 1.3 million deaths were attributed to viral hepatitis, a 200, 000 increase from 2019, with hepatitis B accounting for 1.1 million fatalities, representing 83% of all viral hepatitis-related deaths. The Polaris Observatory Collaborations estimated a 7.2% prevalence of HBV infection in China in 2022, corresponding to 79.7 million chronic HBV carriers (World Health Organization, 2024). Consequently, chronic hepatitis B (CHB) remains a significant public health concern in China and globally. Clinical research and practice have demonstrated that effective antiviral therapy using potent nucleos(t)ide analogs (NAs) with a high genetic barrier to resistance can reverse liver fibrosis, prevent liver-related complications, and improve patient survival (Buti et al., 2018). This approach is a crucial strategy for achieving the World Health Organization’s 2030 goal of eliminating viral hepatitis and promoting clinical cure in CHB patients. “Clinical cure, “ also referred to as functional cure, is defined by sustained HBsAg negativity following treatment cessation, with or without the emergence of anti-HBs, HBV DNA levels below the lower limit of detection, and normal liver biochemical parameters, while cccDNA may persist within hepatocytes (Chinese Society of Hepatology, Chinese Medical Association, 2022). This represents the optimal outcome in the management of CHB. However, with the increasing sensitivity of nucleic acid detection methods, some patients previously considered to have achieved a Complete Virological Response (CVR) after long-term, regular treatment with first-line NAs, still exhibit detectable HBV DNA levels above the current detection limits (Lu et al., 2021).

Comprehensive research reports on the mechanisms underlying LLV in CHB patients, the correlation between LLV and long-term clinical outcomes, the management and monitoring of this patient population, and the latest hepatitis B treatment drugs are currently limited. Both domestic and international guidelines and expert consensus consider LLV a specific condition of CHB, primarily focusing on definitions and treatment recommendations. Comprehensive reviews of its epidemiology, risk factors, and prognostic analyses are relatively scarce. This article aims to review the research progress in this field, explore the challenges and shortcomings, and look forward to future research directions to provide new insights for clinical practice and help improve the long-term prognosis and treatment outcomes of LLV patients.

## 2 Definition and epidemiology of LLV

According to the Chinese Guidelines for the Prevention and Treatment of Chronic Hepatitis B (2022 edition), patients with CHB who are receiving entecavir (ETV), tenofovir disoproxil fumarate (TDF), tenofovir alafenamide fumarate (TAF), or tenofovir amibufenamide (TMF) treatment and demonstrate good adherence, and in whom HBV DNA remains detectable after 48 weeks or more of therapy using high-sensitivity quantitative PCR (with a detection limit typically between 10–20 IU/ml), yet remains below 2000 IU/ml, are defined as having LLV (Chinese Society of Hepatology, Chinese Medical Association, 2022). Persistent low-level viremia is defined as HBV DNA levels consistently between 12 IU/mL and 1999 IU/mL throughout the entire follow-up period. Conversely, intermittent low-level viremia is characterized by the presence of intermittent detectable HBV DNA (12 IU/mL to 1999 IU/mL) in serum, despite achieving complete virological response (Lu et al., 2021). With advancements in detection methodologies, LLV can be further categorized based on viral load, specifically into LLV (20–2000 IU/mL) and very low-level viremia (VLLV) (10–19 IU/mL) (Zhang et al., 2021a). Currently, research on intermittent LLV, persistent LLV, and VLLV is limited. The distinctions between these conditions and LLV concerning viral replication characteristics, hepatic inflammation markers, and clinical outcomes, such as the incidence of adverse prognoses, remain unclear. Further in-depth investigation is warranted to elucidate these differences.

A clinical study conducted from 2017 to 2019 enrolled 517 patients treated with ETV for over 48 weeks, with LLV observed in 180 patients, representing more than 30% of the cohort (Chen et al., 2024). Another large retrospective study included 674 CHB patients who received antiviral therapy for more than 12 months between 2006 and 2020, revealing LLV in 203 patients (30.12%) (Zhang et al., 2021b). A 2020 Chinese longitudinal cohort study included 163 patients treated with ETV-based therapy for 78 weeks, with HBV DNA levels assessed at week 78 (lower limit of detection, 20 IU/mL). The results indicated that LLV was still detectable in 23% of patients (Sun et al., 2020). A large retrospective study from South Korea, encompassing 875 treatment-naïve CHB patients who received ETV monotherapy for over 12 months between 2007 and 2012, observed LLV in 377 patients, representing over 40% (Kim et al., 2017). These findings underscore the necessity for refining long-term monitoring and management strategies for CHB patients in clinical practice. Specifically, further investigation is warranted to determine the optimal timing for implementing highly sensitive detection techniques, facilitating early identification and precise diagnosis of LLV.

## 3 Pathogenesis of LLV

The mechanisms underlying LLV in CHB remain incompletely elucidated. The development of LLV likely involves a confluence of factors, including the limitations of nucleos(t)ide analogs in competitively inhibiting viral replication, the persistence of HBV cccDNA, inadequate host immune responses, the low proliferative state of hepatocytes, and host genetic predispositions.

### 3.1 The persistence of HBV cccDNA and the limitations of NAs in suppressing viral replication

HBV initiates infection by attaching to heparan sulfate proteoglycans (HSPGs) via specific loops within its envelope proteins, followed by binding to the sodium taurocholate co-transporting polypeptide (NTCP) on the basolateral membrane of hepatocytes, facilitating cellular entry. Subsequently, the viral nucleocapsid delivers the HBV relaxed circular DNA (rcDNA) into the hepatocyte nucleus, where it is converted to covalently closed circular DNA (cccDNA) by the host DNA repair machinery. Simultaneously, pregenomic RNA (pgRNA), transcribed from cccDNA, serves as a template for reverse transcription, generating new rcDNA (Wang et al., 2020). On one hand, the newly synthesized rcDNA can be packaged into complete viral particles to infect new, healthy hepatocytes. On the other hand, rcDNA can also enter the nucleus and, after repair, complement the cccDNA within the nucleus, thereby maintaining the stability of the cccDNA pool within the hepatocyte nucleus (Kapoor and Kottilil, 2014). The primary mechanism of NAs involves competitive binding with the HBV polymerase protein within the cell, thereby preventing the entry of rcDNA into the nucleus and inhibiting the synthesis of progeny virus rcDNA. Consequently, NAs do not directly affect cccDNA, which stably resides in the nuclei of infected hepatocytes in a minichromosome form. Furthermore, in the presence of abundant dNTPs, NAs cannot completely suppress HBV replication or prevent the formation of cccDNA in newly infected hepatocytes (Lu et al; Koumbi, 2015; Boglione et al., 2024). The limitation of NAs is evident in their inability to directly eliminate cccDNA, which is stably present within the nuclei of infected hepatocytes, thus hindering the fundamental cure of HBV infection.

### 3.2 Host immune dysfunction

(1) HBV Evasion of Innate Immune Recognition: The natural history of CHB infection is dynamic, with the clinical course categorized into five phases based on HBsAg and HBeAg status, HBV DNA levels, and ALT levels. The first phase is the immune tolerance phase. During this phase, HBeAg is positive, with high-level expression of HBV DNA and HBsAg, while ALT remains persistently normal. This indicates an absence of immune-mediated liver damage during the initial stages of viral infection. Consequently, the hepatitis B virus has evolved a unique replication strategy, potentially involving nuclear sequestration of the transcriptional template, active evasion of innate immune recognition, or active interference with innate immune signaling pathways, thereby inducing immune suppression (Trépo et al., 2014).In a study involving 105 treatment-naïve CHB patients, a significant downregulation of antiviral effector genes, interferon-stimulated genes, Toll-like receptors, and pathogen recognition receptor pathway genes was observed in the liver compared to healthy controls. This suggests a severe impairment of hepatic innate immune function in CHB patients. This impairment may lead to a significant reduction in the ability to recognize and clear the virus, thereby facilitating the long-term persistence of HBV. Furthermore, the chronic presence of the virus can induce immune tolerance, weaken adaptive immune responses, and potentially trigger a chronic inflammatory state, further exacerbating liver damage and disease progression (Lebossé et al., 2017).In a study of clinically cured CHB patients, distinct hepatic gene expression profiles were observed compared to those with unresolved CHB. The research revealed significant upregulation of immune-related genes, including HLA-DPB1 and SERPIN-E1, in clinically cured patients. Specifically, the upregulation of genes involved in immune pathways, such as antigen processing, presentation, and chemokines, suggests that the restoration of hepatic immune function contributes to viral clearance following clinical cure. Conversely, patients with unresolved CHB exhibited significant downregulation of immune gene expression, reflecting a persistent state of immune suppression (Jansen et al., 2015).Compromised host immune function creates conditions conducive to persistent HBV infection, which may represent one of the underlying mechanisms for LLV occurrence.

(2) T cell dysfunction: In CHB patients, persistent antigenic stimulation can lead to immune exhaustion and impaired immune responses in CD8+ T cells. Studies have demonstrated that CHB patients exhibit significantly reduced proliferative capacity and diminished production of IFN-γ, IL-2, TNF-α, granzyme, and perforin by CD8+ effector T cells. This impairment compromises their antiviral function, thereby promoting the sustained replication of cccDNA and HBV, and perpetuating hepatocyte infection (Iannacone and Guidotti, 2022).Furthermore, under the persistent antigenic stimulation characteristic of chronic infection, the effective development of memory T cells is also impaired. This leads to a progressive loss of effector function, manifesting as phenotypic alterations, dysregulation of expansion and differentiation, and ultimately, immune exhaustion. Exhausted T cells exhibit elevated expression of inhibitory receptors, which impedes viral clearance and the restoration of hepatic immune function (Wang et al., 2023).Moreover, to assess the impact of antiviral therapy on the host immune response in HBV-infected patients, we evaluated a cohort of patients with virological suppression undergoing long-term treatment (median treatment duration, 108 months). We observed a negative correlation between serum HBsAg levels and the overall magnitude of virus-specific T cell responses. Notably, patients with HBsAg levels <1000 IU/mL exhibited significantly higher overall response magnitudes, as well as enhanced anti-core immune responses (Loggi et al., 2013).This indicates that persistent T cell dysfunction exists in CHB patients, resulting in impaired viral clearance and subsequent development of LLV.

However, an *in vitro* study revealed that in patients undergoing NAs treatment with HBV DNA suppression but persistent HBsAg, T-cell responses were significantly lower than in patients with complete infection control, yet significantly higher than in treatment-naïve CHB patients and chronic inactive HBsAg carriers with undetectable serum HBV DNA. Notably, the percentage of T cells secreting IFN-γ, IL-2, and TNF-α within this patient cohort did not increase with decreasing HBsAg levels. Despite the limitations inherent in *in vitro* experiments, this study prompts consideration of whether the restoration of T-cell function is invariably correlated with significant changes in serum antigen levels (Le Bert et al., 2020).

**Figure 1:**
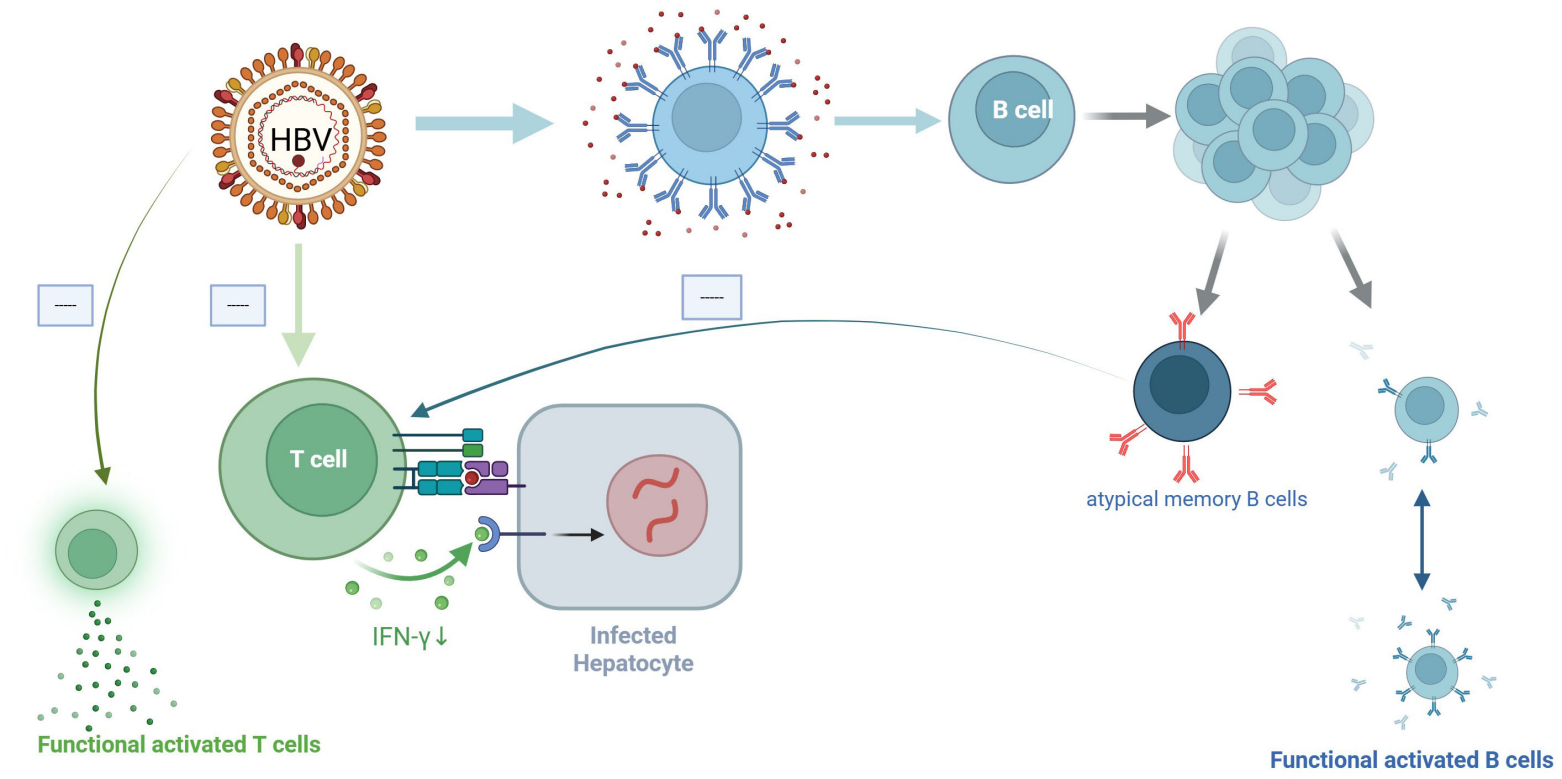
Effects of HBV on T cells and B cells.

(3) B cell dysfunction: Sustained HBsAg stimulation leads to the exhaustion of specific T cells, while its impact on B cells remains less explored. In CHB patients, B cells contribute to antigen presentation and antibody production, potentially interacting with phagocytes or cytotoxic cells to eliminate infected cells. Furthermore, they secrete cytokines, which can also affect infected cells (Bertoletti and Ferrari, 2016).In CHB patients, while HBsAg-specific B cells persist, their antibody-producing capacity is impaired. An experiment involving 84 CHB patients demonstrated the presence of detectable B cells, yet antibody levels remained undetectable (<10 IU/ml). In contrast, healthy individuals vaccinated against HBV exhibited robust *in vitro* differentiation of HBsAg-specific B cells, yielding substantial anti-HBs levels (>1000 IU/ml) (Burton et al., 2018).Additionally, in CHB patients, atypical memory B cells are generated, which differ from normal memory B cells. These cells exhibit defects in signal transduction, survival, antiviral cytokine production, and differentiation into antibody-producing cells (Zheng et al., 2022).This leads to reduced secretion of cytokines such as TNF-α, IFN-γ, and IL-6, which act on cccDNA. Consequently, cccDNA cannot be effectively cleared, potentially contributing to the development of LLV in CHB patients the effects of HBV on T cells and B cells are shown in (**Figure 1**).

(4) Imbalance in Th1/Th2 immune responses: As is well-established, a Th1/Th2 ratio skewed towards Th2 cells is considered detrimental to viral clearance (Fisicaro et al., 2020).Research indicates that HBeAg can drive the host’s immune response towards a Th2 response, which is incapable of clearing the virus, thereby enabling viral survival and successful immune evasion. Furthermore, patients with CHB exhibit a significantly elevated proportion of peripheral blood Treg cells compared to healthy individuals. Treg cells play a crucial role in maintaining immune tolerance, suppressing the activation and proliferation of T lymphocytes, and inhibiting the maturation of dendritic cells. In addition, Tregs can also suppress the capacity of T follicular helper cells through the expression of CTLA-4, leading to their inability to assist B cell function maturation and the production of neutralizing antibodies, thus contributing to persistent HBV infection (Wing et al., 2014).

(5) Natural Killer cell(NK cell) and Dendritic cell(DC cell) dysfunction: Multiple studies have confirmed that NK cell function is universally impaired in chronic hepatitis B patients, characterized by reduced cytotoxicity (including decreased expression of granzyme B and perforin), diminished secretion of antiviral cytokines (such as IFN-γ and TNF-α), and upregulated expression of inhibitory receptors (including NKG2A and Tim-3) (Li et al., 2017). This functional impairment correlates with high viral loads, whereas NK cell function may partially recover when viral loads are reduced (Zimmer et al., 2018).Impaired NK cell function may indirectly facilitate LLV persistence, manifesting in the following aspects: First, this leads to the formation of an immunosuppressive microenvironment. In CHB patients, HBV antigens can induce regulatory T cells (Tregs) to secrete IL-10, which subsequently upregulates the inhibitory receptor NKG2A on NK cell surfaces, thereby attenuating their antiviral activity (Ma et al., 2020). This immunosuppressive state may impede complete viral clearance, resulting in LLV. Furthermore, chronic HBV infection leads to NK cell expression of exhaustion markers (such as PD-1 and Tim-3), diminishing their responsiveness to viral antigens (Selim et al., 2024).Even at low viral loads, exhausted NK cells may fail to effectively eliminate residual virus. Furthermore, NK cell subsets (such as CD56bright and CD56dim) exhibit distinct behaviors in chronic hepatitis B. The CD56bright subset demonstrates enhanced cytokine production capacity; however, its functional restoration may remain incomplete in patients with low-level viremia. Conversely, the CD56dim subset shows more pronounced impairment in cytotoxic function (Gu et al., 2021), and this imbalance may compromise the efficiency of viral control mechanisms. Finally, HBV-induced suppressive monocytes drive NK cell differentiation toward regulatory NK cells (NK-regs). These NK-regs express anti-inflammatory cytokines, including interleukin-10 (IL-10) (Li et al., 2018). Elevated IL-10 levels establish an immunosuppressive cytokine milieu that may impair NK cell interferon-γ production, thereby compromising the efficiency of viral replication cycle inhibition (Li et al., 2019). Although NK cell function correlates with viral control, it exerts bidirectional regulatory effects on chronic hepatitis B virus infection: hyperactivated NK cells may exacerbate hepatic injury (Jin and Bi, 2022), while hypofunction facilitates viral persistence. Therefore, the optimal therapeutic goal is “moderate restoration” of NK cell function to achieve a balance between viral clearance and immunopathological damage.

DCs are specialized antigen-presenting cells responsible for processing antigens and presenting them to T cells, while also participating in the production of cytokines that influence T cell polarization. Studies have demonstrated that the number of plasmacytoid dendritic cells (pDCs) in CHB patients is significantly lower than in healthy controls (Jin et al., 2021). Monocyte-derived dendritic cells from hepatitis B patients exhibit impaired function, resulting in diminished T cell production of interleukin-2 (IL-2), TNF-α, and IFN-γ (Beckebaum et al., 2003).Concurrently, studies have demonstrated that pDCs in chronic hepatitis B patients exhibit impaired capacity to activate CD4+ T cells (Hong and Gong, 2008). Myeloid dendritic cells (mDCs) derived from CHB patients demonstrated reduced efficacy in inducing T cell proliferation *in vitro* compared to mDCs isolated from healthy individuals (Chen et al., 2007).Additionally, patients with high viremia exhibit reduced OX40 ligand expression in peripheral blood, and virus carriers show decreased OX40L expression on pDCs activated by TLR9-L, which blocks their capacity to induce natural killer (NK) cell cytotoxic activity, consequently resulting in impaired NK cell function (Martinet et al., 2012).

HBV infection induces functional impairment in dendritic cells and natural killer cells, manifested by suppressed receptor expression, diminished cytotoxicity, and an immunosuppressive cytokine profile. Therapeutic interventions achieve only partial restoration of immune cell function (Shu et al., 2025). These functional deficits in immune cells and their incomplete recovery following treatment may represent one of the underlying mechanisms contributing to low-level viremia.

### 3.3 Hepatocellular quiescence

Multiple studies have demonstrated that in primary human hepatocytes infected with HBV, the expression levels of all virological markers, including cccDNA copy number, rapidly decline during periods of rapid compensatory proliferation. Conversely, as hepatocyte compensatory proliferation slows, virological markers begin to rebound (Mason et al., 2010).*In vitro* studies have demonstrated that the proliferation of human hepatocytes can significantly reduce HBV cccDNA. This research utilized humanized mice (uPA/SCID/beige) transplanted with primary human hepatocytes (PHH) infected with HBV. Serial transplantation induced hepatocyte proliferation, revealing a significant reduction in whole-liver cccDNA. The mechanism appears to involve dilution (unequal distribution of cccDNA during cell division) and loss (absence of centromeric structures), thereby decreasing cccDNA. Notably, this process did not detect significantly elevated pro-inflammatory factors, suggesting that cccDNA clearance primarily relies on proliferation itself rather than inflammation-mediated immune mechanisms (Allweiss et al., 2018).Furthermore, *in vitro* studies have revealed that HepG2-NTCPsec cells, infected with HBV but exhibiting slow replication, release progeny virions with enhanced infectivity. This suggests that when hepatocytes are in a state of low proliferation, they secrete more infectious viral particles, thereby promoting HBV reinfection (König et al., 2019).

In clinical practice, patients exhibiting significantly elevated ALT levels demonstrated a marked reduction in serum HBV DNA load during NAs antiviral therapy. The group with higher inflammatory activity exhibited a superior virological response to antiviral treatment, indicating that patients with active hepatic inflammation are more likely to achieve complete virological response (CVR) during NAs antiviral therapy (Wong et al., 2018).In a clinical study, patients exhibited a more pronounced decline in serum HBV DNA load in conjunction with elevated ALT levels, indicative of hepatocyte damage, during NAs therapy (Yan et al., 2019).Furthermore, patients with heightened hepatic inflammatory activity at baseline demonstrated a more significant reduction in serum HBV DNA and HBeAg levels following six months of antiviral treatment, compared to those without significant liver inflammation (Guan et al., 2021).

The *in vitro* findings contrast with clinical observations in CHB patients: While NAs effectively suppress HBV DNA and reduce hepatic inflammation, they may indirectly support the persistence of LLV by potentially delaying cccDNA clearance due to decreased hepatocyte proliferation.

In summary, while NAs effectively inhibit HBV DNA synthesis, they do not directly eliminate cccDNA, the intrahepatic HBV reservoir, thereby precluding a functional cure for CHB patients with monotherapy. Furthermore, chronic exposure to HBsAg impairs immune cell function and phenotype, leading to reduced cytokine secretion and supporting the progression to LLV. Moreover, in the context of low viral loads and diminished hepatic inflammation during chronic infection, suppressing viral replication alone may be insufficient for complete cccDNA clearance. Therefore, combination strategies are warranted, such as promoting hepatocyte regeneration combined with antiviral agents to block reinfection or replication, disrupting the vicious cycle of persistent viral infection, and accelerating cccDNA clearance. Further research should explore the synergistic effects of immunomodulators to enhance hepatocyte proliferation and cccDNA clearance, as well as develop methods to specifically eliminate non-replicating, persistently infected hepatocytes.

Risk Factors for Low-Level Viremia in CHB Patients(Summary of LLV risk factors and potential mechanisms is shown in **Table 1**).

**Table 1:** Summary of LLV risk factors and potential mechanisms.

Risk factors	Potential mechanisms	References
Use of non-first-line nucleoside analogs *	No direct effect on ccc DNA that stably exists in the form of minichromosomes within the nuclei of infected hepatocytes.	(Zhang et al., 2021b)
Higher baseline HBV DNA levels*	High viral load may reflect a larger viral reservoir size, leading to poor treatment response.	(Zhang et al., 2021b; Chen et al., 2024; Li et al., 2024)
Higher HBV DNA levels after 6 months of treatment*		(Zhang et al., 2021b)
The initial achievement of CVR occurred at a relatively late time point.		(Kim et al., 2017)
HBeAg positive at baseline*	HBeAg can promote the transcription of pg RNA by regulating viral promoter activity, thereby maintaining the stability of the ccc DNA pool.	(Kim et al., 2017; Chen et al., 2024; Li et al., 2024)
Steatotic liver consolidation*	The intracellular accumulation of lipid droplets in hepatocytes leads to reduced bioavailability of NA metabolites in the liver.	(Jin et al., 2012; Jiang et al., 2021; Zhang et al., 2023; Li et al., 2024)

*Non-first-line nucleoside analogs: Lamivudine, adefovir dipivoxil, telbivudine, and other low-resistance second-line drugs;* High HBV DNA level: refers to HBV DNA >2*10^5 IU/ml; *High HBV DNA level at 6 months of treatment refers to HBV DNA ≥3 log 10 IU/L; *CVR achievement time: Achieving CVR within 1 year is defined as early CVR achievement; beyond 2 years is defined as late CVR achievement; *HBeAg positive: semi-quantitative detection >1.0 COI; *Definition of fatty liver: refers to the pathological state of abnormal accumulation of triglycerides (TG) in hepatocytes.

With advancements in HBV DNA detection methodologies, there’s a growing body of research concentrating on patients exhibiting LLV. Prior studies have largely focused on identifying risk factors by comparing baseline characteristics and laboratory parameters between LLV patients and those achieving sustained virological response (SVR).

## 4 Currently identified risk factors for LLV

A clinical study conducted from 2017 to 2019 enrolled 517 patients treated with ETV for over 48 weeks; 180 of these patients developed LLV. Multivariate analysis revealed that pre-treatment baseline HBeAg positivity, high HBV DNA levels, and high HBsAg levels were significantly associated with the risk of LLV after long-term antiviral therapy with ETV (Chen et al., 2024). A retrospective study in China, including 674 CHB patients who received antiviral therapy for more than 12 months between 2006 and 2020, observed LLV in 203 patients (30.12%). Multivariate analysis identified non-first-line drug treatment, lower baseline ALT, higher baseline HBV DNA levels, and HBV DNA levels at 6 months as independent risk factors for LLV (Zhang et al., 2021b). Another study conducted in China enrolled 1653 patients undergoing ETV treatment from 2018 to 2023. Of these, 1181 patients (71.4%) achieved CVR, while 472 patients (28.6%) experienced LLV. Multivariate analysis revealed that HBeAg positivity, baseline HBV DNA ≥ 6.0 Log10 IU/mL, HBsAg ≥ 9000 IU/mL, cirrhosis, PLT < 100×109/L, and LSM ≥ 13.0 kPa at baseline (all *p* < 0.05) were independent risk factors associated with LLV in CHB patients following long-term ETV treatment (Li et al., 2024). A retrospective study conducted in South Korea analyzed 875 treatment-naïve adult CHB patients who received ETV monotherapy for over one year between 2007 and 2012. In a multivariate model, HBeAg status was the sole factor associated with LLV. The timing of the first CVR demonstrated a borderline association, with patients achieving their first CVR later (first CVR after two years) more likely to experience LLV compared to those achieving their first CVR earlier (within one year) (Kim et al., 2017).

## 5 Fatty liver disease represents a controversial risk factor for LLV

### 5.1 Adverse effects on viral response

In a recent study conducted in China, we investigated the detrimental impact of nonalcoholic fatty liver disease (NAFLD) on the efficacy of CHB treatment. Our findings revealed that, among treatment-naïve CHB patients enrolled between 2015 and 2021, those with concurrent NAFLD experienced a delay in achieving CVR and exhibited a higher incidence of LLV, the combined incidence of LLV was 48.8%. These observations suggest that NAFLD may also serve as a significant risk factor for LLV (Zhang et al., 2023).A prospective study conducted in China enrolled 213 treatment-naïve CHB patients receiving ETV. Compared to patients with concomitant hepatic steatosis, those with CHB alone demonstrated significantly increased HBV DNA clearance rates at weeks 24, 48, and 96. Furthermore, multivariate logistic regression analysis revealed that hepatic steatosis was an independent predictor of treatment failure at weeks 24, 48, and 96 of ETV therapy (Jin et al., 2012).Similarly, another study including 465 CHB patients from 2011–2022 demonstrated that during 24 months of NAs therapy, the NAFLD group exhibited slower decline in HBV DNA levels and significantly lower rates of HBV DNA negativity compared to the non-NAFLD group (Li et al., 2024). Another meta-analysis conducted in China, focusing on CHB patients with concomitant hepatic steatosis (HS), demonstrated that the presence of HS negatively impacts the virological response in treatment-naïve CHB patients (Jiang et al., 2021).This may be attributed to reduced bioavailability of intrahepatic NA metabolites due to lipid droplet accumulation within hepatocytes (Taliani et al., 1995), resulting in diminished antiviral efficacy of NAs. Additionally, decreased hepatocyte cytochrome activity in steatotic hepatocytes may impair drug metabolism, thereby adversely affecting therapeutic response (Leclercq et al., 1998).

### 5.2 No significant impact on viral response

A meta-analysis retrieving 2, 108 studies found no significant differences in biochemical response rates, complete viral suppression rates, or HBeAg seroconversion rates between CHB patients with concurrent hepatic steatosis and those with CHB alone following antiviral therapy for up to 48 or 96 weeks (Rui et al., 2024).Similarly, in a study encompassing 555 CHB patients from 2000 to 2016, no statistically significant differences were observed in biochemical response rates and complete viral suppression rates between CHB patients with concurrent NAFLD and those with CHB alone (Li et al., 2020).These studies suggest that concurrent fatty liver disease has no significant impact on virological response in treatment-experienced CHB patients and shows no correlation with LLV development.

### 5.3 Beneficial for reducing HBV DNA and HBsAg levels

Interestingly, a large-scale Hong Kong study involving 1202 patients revealed that untreated CHB patients with metabolic dysfunction-associated steatotic liver disease (MASLD) exhibited lower HBV DNA levels compared to those without MASLD. Furthermore, HBV DNA levels decreased with increasing severity of steatosis. However, in patients undergoing NAs treatment, no significant differences were observed between CHB patients with and without MASLD. Notably, the cumulative incidence of HBsAg seroclearance was higher in the CHB-MASLD group compared to the CHB group and those without MASLD (Hui et al., 2018).In a study involving 4, 084 treatment-naïve CHB patients, 21.7% were found to have co-existing MASLD. CHB patients with MASLD and ≥3 metabolic criteria (including central obesity, elevated blood pressure, hypertriglyceridemia, low high-density lipoprotein cholesterol, and abnormal glucose) exhibited the highest HBsAg clearance rates (Huang et al., 2024).A prospective study involving 53 treatment-naïve CHB patients demonstrated lower quantitative HBsAg levels in patients with coexisting MASLD compared to those without metabolic factors (Patel et al., 2024).Furthermore, several other studies have indicated that the presence of hepatic steatosis contributes to HBsAg clearance in CHB patients (Chu et al., 2007).This could be attributed to the synergistic effects of metabolic disorders, such as chronic inflammation and adipokine imbalance, associated with coexisting MASLD, potentially triggering a more robust immune response and thus facilitating HBsAg clearance, whereas the latter may not have reached the threshold for immune activation (Breuer et al., 2020).Additionally, it may potentially reduce HBsAg clearance through activation of innate immune responses (such as enhanced antigen presentation or NK cell activity) (Zhang et al., 2015; Liu et al., 2023), thereby altering metabolism (such as downregulation of PGC-1αexpression) (Shlomai et al., 2006; Piccinin et al., 2019). Studies have also demonstrated that hepatocyte steatosis can suppress HBsAg and HBV DNA secretion through induction of endoplasmic reticulum stress in hepatocytes (Liu et al., 2023).However, it is noteworthy that the aforementioned studies predominantly involved treatment-naïve patients, and more extensive and comprehensive investigations are warranted regarding the occurrence of LLV in nucleos(t)ide analogue-experienced CHB patients with concurrent NAFLD.

The aforementioned research underscores the intricate relationship between virological response and immune status in CHB patients coexisting with NAFLD or MASLD. The direction of this association may exhibit significant variability, contingent upon the study population, the specific type of steatotic liver disease, or the degree of metabolic dysfunction. In clinical practice, individualized therapeutic strategies are essential for CHB patients with comorbid metabolic abnormalities, such as obesity and insulin resistance. Optimization of virological monitoring protocols, including the use of high-sensitivity HBV DNA assays and dynamic HBsAg quantification, along with tailored follow-up intervals, is recommended. Furthermore, the mechanisms underlying the interaction between NAFLD or MASLD and LLV remain incompletely elucidated. Further investigation is warranted to determine their respective contributions to virological breakthrough or sustained low response, necessitating prospective cohort studies or randomized controlled trials. Particular attention should be given to the potential regulatory effects of metabolic interventions, on the risk of LLV.

In summary, recent investigations into LLV development in CHB patients have identified several potential influencing factors, including: 1) utilization of non-first-line nucleos(t)ide analogs; 2) lower baseline ALT levels; 3) elevated baseline HBV DNA and HBeAg levels; 4) higher HBV DNA levels at 6 months of treatment; 5) presence of cirrhosis; 6) delayed achievement of CVR; and 7) co-occurrence of hepatic steatosis. Despite the valuable insights these studies offer regarding the prediction and management of LLV, several knowledge gaps and challenges persist. Firstly, while multiple studies have identified risk factors for LLV, the precise immunological mechanisms and detailed processes of virus-host interactions remain incompletely understood. Further validation is required to accurately assess the contribution of these factors to LLV occurrence and their applicability across diverse populations. Secondly, current research predominantly focuses on patient cohorts undergoing specific antiviral therapies, with limited investigation into the impact of other antiviral agents or combination therapies. Future studies should examine the differences in LLV incidence across various treatment regimens and further explore how to mitigate the risk of LLV through personalized therapeutic strategies.

## 6 Impact of LLV on the prognosis of CHB

LLV may impact the prognosis of patients with CHB. Alterations in the hepatic microenvironment in LLV patients, coupled with host-HBV interactions, may contribute to liver damage, correlating with the progression of liver fibrosis and the development of hepatocellular carcinoma.

### 6.1 Increased risk of liver fibrosis progression.

In a recent study conducted in China, we employed various non-invasive methods to comparatively analyze 92 CHB patients undergoing >1 year of NAs monotherapy from 2019 to 2023. The findings revealed that at both 72 and 96 weeks, the LLV group exhibited significantly higher FIB-4 scores compared to the SVR group (P<0.05 for both time points). This trend suggests that LLV may contribute to the progression of liver fibrosis (Xu et al., 2024). In a separate longitudinal study conducted in China, involving 239 paired liver biopsies from CHB patients, the findings indicated that detectable HBV DNA at 78 weeks of treatment and alcohol consumption were independent risk factors for the progression of liver fibrosis. After excluding alcohol consumption, the detection rate of HBV DNA at 78 weeks in the liver fibrosis progression group (50%) remained significantly higher than in the liver fibrosis regression group (19%) or the indeterminate group (26%), despite low-level viremia (20–200 IU/mL) across all groups. This suggests that LLV at 78 weeks of treatment is associated with fibrosis progression, emphasizing the need for enhanced HBV DNA monitoring (Sun et al., 2020).

A study of 674 CHB patients who received antiviral therapy for more than 12 months between 2006 and 2020 revealed that the cumulative incidence of end-stage liver disease at 5 and 10 years was significantly higher in LLV patients than in SVR patients during a median follow-up of 42 months (5 years: 7.73% vs. 0.77%; 10 years: 15.85% vs. 5.52%). The risk of HCC was higher in LLV patients in the high-risk group of the four HCC risk models (*p* < 0.05). Cox regression analysis indicated that LLV was an independent risk factor for end-stage liver disease and HCC (Zhang et al., 2021b). Furthermore, regarding the correlation between cirrhosis and HCC, a prospective study revealed that patients exhibiting a reduction in liver stiffness measurement (LSM) during follow-up demonstrated a lower risk of developing HCC compared to those with persistently elevated LSM (Jung et al., 2011).

The findings from this study underscore the significant impact of low-level viremia (LLV) on the progression of liver fibrosis. LLV may contribute to chronic inflammation and hepatic damage through persistent, low-level HBV viremia, thereby accelerating the fibrotic process. Furthermore, LLV is closely associated with the development of end-stage liver disease and HCC.These results emphasize the importance of carefully assessing the risk of liver fibrosis in patients with LLV within clinical management protocols. Enhanced monitoring of HBV DNA levels and the exploration of optimized antiviral treatment strategies are warranted to mitigate the risk of long-term hepatic complications.

### 6.2 The incidence of HCC is elevated in CHB

A multicenter, retrospective study conducted in South Korea enrolled 3, 624 treatment-naïve CHB patients who were monitored between 2006 and 2011. The study revealed that the 5-year cumulative incidence of HCC was 13.9% in CHB patients with LLV and concurrent cirrhosis, compared to 1.7% in CHB patients with LLV alone (Sinn et al., 2019). Another retrospective cohort study conducted in South Korea analyzed 875 CHB patients initiating ETV monotherapy between 2007 and 2012, evaluating HCC development during follow-up. The study compared HCC risk between patients achieving sustained virological response (SVR) and those with LLV. With a median follow-up of 4.5 years, the LLV group exhibited a 1.98-fold increased risk of HCC compared to the SVR group. Furthermore, among patients with cirrhosis, the HCC risk in the LLV group was 2.20 times higher than in the SVR group (Kim et al., 2017). A retrospective study conducted in China, involving 674 CHB patients treated with oral nucleos(t)ide analogs (NAs) between 2006 and 2020, similarly demonstrated that patients with LLV exhibited a significantly elevated risk of developing end-stage liver disease (decompensated cirrhosis and HCC) at both 5 and 10 years compared to those SVR(*p* < 0.050) (Zhang et al., 2021b). In a separate study, 565 CHB patients diagnosed with HCC and concurrent LLV between 2010 and 2013 were analyzed. The findings indicated that HBV-related HCC patients with LLV experienced frequent HBV reactivation during follow-up and exhibited poorer overall survival compared to the SVR group (Kim et al., 2019).A Taiwanese study, encompassing 16, 895 CHB patients from 2008 to 2020, reported 408 incident HCC cases during a median follow-up of 5.78 years. The cumulative HCC risks at 3, 5, and 10 years were 3.56%, 4.96%, and 9.51% in the LLV cohort, respectively. Independent risk factors for HCC development in the LLV group included male gender, advanced age, cirrhosis, and lower platelet counts (Chen et al., 2024).

The findings from this study demonstrate that LLV significantly elevates the risk of HCC development in patients with CHB. Across both treated and untreated cohorts, the cumulative incidence of HCC was notably higher in patients exhibiting LLV compared to those achieving SVR. This increased risk was particularly pronounced in patients with concomitant cirrhosis, where the likelihood of HCC occurrence was further amplified.

## 7 Management and treatment of patients with LLV

LLV significantly impacts the long-term prognosis of CHB patients. Management and treatment strategies for LLV patients necessitate a precise assessment of disease risk, optimization of antiviral therapy, and enhanced disease monitoring to mitigate the risk of liver fibrosis, HCC, and end-stage liver disease. Furthermore, the development of novel therapeutic approaches and the refinement of risk prediction models offer crucial avenues for personalized management of LLV patients. For CHB patients undergoing long-term treatment, high-sensitivity HBV DNA testing, along with assessments of HBsAg, HBeAg, anti-HBs, anti-HBe, biochemical parameters, and platelet counts, should be conducted every 3–6 months. Subsequent treatment decisions should be based on these laboratory findings.

### 7.1 Management recommendations proposed by different studies:

#### 7.1.1 Modify the existing drug dosage

A 2021 study examining ETV investigated the correlation between ETV dosage and efficacy by analyzing all available HBV treatment articles, prescription information for NAs, and literature on LLV from the MEDLINE/PUBMED databases and the U.S. Food and Drug Administration (FDA) website over nearly 25 years. The study integrated data from clinical, pharmacokinetic, and pharmacodynamic studies, as well as Bergman reviews, and recommended that increasing the entecavir dosage to 1.0 mg/d is feasible and associated with a favorable prognosis for treatment-naïve patients with HBV DNA >2 × 10^6 IU/mL. Further research is warranted to assess the long-term toxic effects of high-dose ETV (2.5mg/d), which may prove beneficial for patients previously treated with NAs, those with partial virological response, or those in an LLV state (Yin et al., 2021).

#### 7.1.2 Switching therapeutic agents

In a study of 674 CHB patients treated with oral NAs between 2006 and 2020, 24 of 203 patients in the LLV group had their treatment regimens altered. The results indicated that patients with adjusted treatment were more likely to achieve complete virological suppression and exhibited superior long-term clinical outcomes (Yin et al., 2021).A multicenter retrospective study conducted in Japan enrolled 313 patients who switched to TAF treatment after receiving ETV or other NAs between 2017 and 2018 to assess their virological response. The results indicated that among the 34 patients with baseline HBV DNA levels of 20–2000 IU/mL, 97.1% achieved HBV DNA <20 IU/mL after 48 weeks of TAF treatment (Ogawa et al., 2020). A prospective study conducted in China analyzed 211 patients treated with ETV between 2018 and 2019. The results demonstrated that at 24 weeks of treatment, the TAF group exhibited significantly higher rates of CVR and ALT normalization compared to the ETV group (CVR: 62.7% vs. 9.3%; ALT: 47.6% vs. 10.5%, *p* < 0.001). The study suggests that switching to TAF is more advantageous than continuing ETV treatment for LLV patients, offering superior virological and biochemical improvements with a favorable safety profile (Li et al., 2021).

### 7.2 International guidelines for LLV treatment management recommendations

The updated EASL clinical guidelines from August 2025 recognize that LLV occurrence is associated with poor adherence and suboptimal enteric absorption of tenofovir (Watkins et al., 2017). Consequently, EASL recommends that TDF and TAF be administered with food to enhance bioavailability, with reinforced patient education in clinical settings. EASL recommends that during TDF, TAF, or ETV therapy, if persistent low-level HBV DNA (<2000 IU/ml) is observed in the absence of advanced hepatic fibrosis and with drug resistance excluded, immediate treatment modification is not warranted (European Association for the Study of the Liver, 2025).

The AASLD chronic hepatitis B virus infection management and treatment algorithm revised in July 2021 continues to employ the definition of partial virological response to characterize patients with HBV DNA levels between 20–2000 IU/ml after 48 weeks of therapy, similarly recommending assessment of medication adherence, though no definitive therapeutic regimen is provided for CHB patients with good compliance (Martin et al., 2022).

The 2022 Chinese guidelines for chronic hepatitis B explicitly defined LLV, excluding patients with poor medication adherence from this definition. The guidelines recommend that LLV patients, after ruling out adherence issues and testing errors, may undergo nucleos(t)ide analogue (NA) therapy adjustment (switching from ETV to TDF or TAF, switching from TDF or TAF to ETV, or combination therapy with two agents), or may receive combination treatment with pegylated interferon-alpha (Chinese Society of Hepatology, Chinese Medical Association, 2022).

From a definitional perspective, EASL attributes LLV occurrence to patient adherence and drug absorption, emphasizing clinical relevance and practical significance, whereas AASLD continues to classify LLV as suboptimal response—a dynamic concept for therapeutic efficacy assessment during treatment that prioritizes drug effectiveness. In contrast, Chinese guidelines proactively exclude confounding factors and provide a clear, unambiguous definition that positions HBV DNA levels as the primary focus requiring heightened attention.

Therefore, regarding subsequent therapeutic management, EASL recommends enhancing patient adherence and administering medications with food to improve bioavailability. In contrast, AASLD provides no definitive recommendations for subsequent treatment after excluding medication non-adherence, similar to EASL, likely due to insufficient high-quality clinical evidence demonstrating clinical benefits for LLV patients following treatment regimen modifications. However, Chinese guidelines adopt a more proactive approach with explicit intervention recommendations, potentially reflecting China’s status as a high hepatitis B burden country and its commitment to advancing the goal of “functional cure” in chronic hepatitis B.

The causes of these discrepancies may stem from varying interpretations of research data across different regions, divergent assessments of long-term LLV risks, and differing therapeutic philosophies regarding treatment objectives. In summary, management and therapeutic strategies for patients with LLV are continuously refined. Treatment adjustments, including dosage modifications, switching, or combination therapies, may be considered in specific cases to improve virological suppression and long-term clinical outcomes. Notably, switching to novel nucleos(t)ide analogs (e.g., TAF) or combination therapies (e.g., ETV+TDF/TAF, Peg-IFN-α) demonstrates significant advantages.

## 8 Summary and outlook

Based on current research gaps, future investigations could explore the following avenues: First, comprehensive viral genome sequencing should be conducted to determine whether specific genetic mutations exist in LLV patients and elucidate how these mutations affect viral replication efficiency and host immune recognition (Zhang et al). Concurrently, host genomic studies should be refined to identify host genetic polymorphisms associated with LLV and clarify their underlying mechanisms of action. Building upon this foundation, integrating genomic variation data from both viral and host organisms to construct virus-host coevolutionary network models will facilitate elucidation of the dynamic equilibrium mechanisms underlying viral escape and host response at the genetic level during low-level viremia, thereby providing a genetic basis for characterizing host-virus interaction networks in LLV.

Second, single-cell RNA sequencing can be performed on hepatic tissue or peripheral blood immune cells from LLV patients to decipher transcriptomic differences across distinct cellular subpopulations, with validation through immunofluorescence analysis. By further integrating viral sequence information, virus-specific T cell clonotypes and their functional states can be identified, thereby constructing a comprehensive virus-immune cell interaction network that elucidates critical nodes of immune evasion in the LLV state. Additionally, quantitative proteomics can be employed to compare protein expression differences between LLV patients and SVR patients, potentially identifying key functional proteins associated with LLV. Combined with metabolomics analysis, this approach may reveal characteristic metabolite alterations in LLV patients. Through protein-metabolite co-expression network analysis, the regulatory relationship between viral replication and host metabolic reprogramming can be further elucidated, expanding our understanding of host-virus interaction networks.

By integrating these multi-omics datasets and combining them with prospective cohort studies (Wang et al., 2024), we anticipate elucidating the core mechanisms underlying LLV occurrence and its impact on CHB progression from a systems biology perspective, thereby providing precise biomarkers, risk stratification, and therapeutic guidance for LLV patients, ultimately advancing the clinical cure of CHB.
